# Characterization of New Defensin Antimicrobial Peptides and Their Expression in Bed Bugs in Response to Bacterial Ingestion and Injection

**DOI:** 10.3390/ijms231911505

**Published:** 2022-09-29

**Authors:** Sanam Meraj, Arshvir Singh Dhari, Emerson Mohr, Carl Lowenberger, Gerhard Gries

**Affiliations:** Department of Biological Sciences, Simon Fraser University, Burnaby, BC V5A 1S6, Canada

**Keywords:** bed bugs, defensin antimicrobial peptides, humoral innate immunity, hematophagy, Toll and IMD pathways, vectors, pathogens, immunization

## Abstract

Common bed bugs, *Cimex lectularius*, can carry, but do not transmit, pathogens to the vertebrate hosts on which they feed. Some components of the innate immune system of bed bugs, such as antimicrobial peptides (AMPs), eliminate the pathogens. Here, we determined the molecular characteristics, structural properties, and phylogenetic relatedness of two new defensins (CL-defensin1 (XP_024085718.1), CL-defensin2 (XP_014240919.1)), and two new defensin isoforms (CL-defensin3a (XP_014240918.1), CL-defensin3b (XP_024083729.1)). The complete amino acid sequences of CL-defensin1, CL-defensin2, CL-defensin3a, and CL-defensin3b are strongly conserved, with only minor differences in their signal and pro-peptide regions. We used a combination of comparative transcriptomics and real-time quantitative PCR to evaluate the expression of these defensins in the midguts and the rest of the body of insects that had been injected with bacteria or had ingested blood containing the Gram-positive (Gr+) bacterium *Bacillus subtilis* and the Gram-negative (Gr–) bacterium *Escherichia coli*. We demonstrate, for the first time, sex-specific and immunization mode-specific upregulation of bed bug defensins in response to injection or ingestion of Gr+ or Gr– bacteria. Understanding the components, such as these defensins, of the bed bugs’ innate immune systems in response to pathogens may help unravel why bed bugs do not transmit pathogens to vertebrates.

## 1. Introduction

The common bed bug, *Cimex lectularius* L., is an obligate hematophagous ectoparasite of humans [1,2] and one of the most challenging indoor pests to eradicate [1,3]. In contrast to many hematophagous arthropods that transmit pathogens to vertebrate hosts, bed bugs are believed not to transmit pathogens to humans [4,5,6], and have been shown to transmit pathogens to vertebrates only under controlled laboratory conditions [7,8,9]. The specific components of their innate immune system that bed bugs activate to eliminate pathogens or parasites are not known.

Humoral immune responses of insects include the production of antimicrobial peptides (AMPs) [10,11,12], reactive intermediates of oxygen or nitrogen [13,14], and the enzymatic cascades that regulate the melanotic encapsulation of parasites and pathogens [15,16]. Initially, it was reported that the Toll signalling pathway responded to Gr+ bacteria and fungi, the immune deficiency (IMD) pathway to Gr– bacteria, and the JAK-STAT and RNAi pathways to viruses [17,18,19,20]. More recent studies have demonstrated that there is plasticity and cross-talk among and between immune pathways that allow strong multifaceted responses to pathogens and parasites [21,22,23,24]. 

The expression of effector molecules such as AMPs regulated by the canonical Toll and IMD pathways has been reported at the transcript level in bed bugs after infection with the Gr– bacterium *Escherichia coli* [25]. The tissue- and pathogen-specific upregulation of effector molecules, and their range of activity and structural characteristics have not been characterized fully in bed bugs. 

Insect AMPs have been categorized as cysteine-rich AMPs [26,27], α-helical AMPs such as cecropins [26,28,29], proline-rich AMPs such as metchnikowins, drosocins, and lebocins [30,31], and glycine-rich AMPs such as the diptericins and attacins [32,33]. Insect defensins are small (<10 kDa), cationic, immune-inducible, cysteine-rich peptides [34]. Defensins are produced as precursor pro-peptides, and characteristically contain three intramolecular disulfide bonds between six conserved cysteine residues that contribute to their stability and antimicrobial activity [34,35]. Defensins are ubiquitous throughout invertebrates [36], with a few exceptions such as the pea aphid, *Acyrthosiphon pisum* [37]. Defensins disintegrate bacterial membranes, interfere with membrane synthesis, and disrupt membrane permeability. They are most strongly active against Gram+ bacteria, fungi, and some protozoan parasites [17,34,38,39]. 

Insect AMPs, including defensins, are expressed principally in the fat bodies of insects and, to a lesser extent, in the midguts, hemocytes, and other insect tissues and organs [34,40,41]. AMPs may be secreted into the hemocoel to eliminate invading pathogens, or into the gut lumen to eliminate ingested pathogens or to break down bacteria during digestion. How insects detect and eliminate invasive pathogens without harming their own obligate microbiota is not fully understood. 

Tissue-specific production of AMPs or their isoforms has been observed after ingestion or injection of bacteria into the insects [42,43]. Some isoforms of AMPs are tissue-specific in their expression, with one isoform being secreted into the gut and another being released into the hemocoel [29,43,44,45]. The kissing bug, *Rhodnius prolixus*, which is a close phylogenetic relative of bed bugs, expresses different defensins and lysozymes in the midgut or fat body dependent upon whether microbes have been ingested or have entered the hemocoel [42,43,46]. The tissue- and pathogen-specific regulation of defensins or other AMPs have not yet been explored in bed bugs. 

Here, we evaluated the expression of insect defensins in the midgut and the rest of the body (RoB) in bed bugs after injection of bacteria into the hemocoel, or ingestion of a blood meal containing bacteria. We describe two new defensins identified in a transcriptome made from immunized insects and characterized their molecular features, structural properties, and phylogenetic relatedness with defensins from other invertebrates. Using comparative transcriptomics and quantitative real-time PCR, we investigated the effects of different bacteria, bed bug tissue, and bed bug sex, and compared the route of entry of bacteria (ingestion vs. injection) on the upregulation of these defensins in bed bugs. Our study adds to the current knowledge about the diversity of AMPs expressed in bed bugs, and it provides a foundation for further research on humoral immunity of bed bugs in response to pathogenic infections. The AMP diversity in bed bugs may be one of multiple factors that might explain why bed bugs do not transmit parasites and pathogens to their vertebrate hosts. 

## 2. Results

### 2.1. Structural and Phylogenetic Analyses of CL-Defensins (Figure 1 and Figure 2)

**Figure 1:**
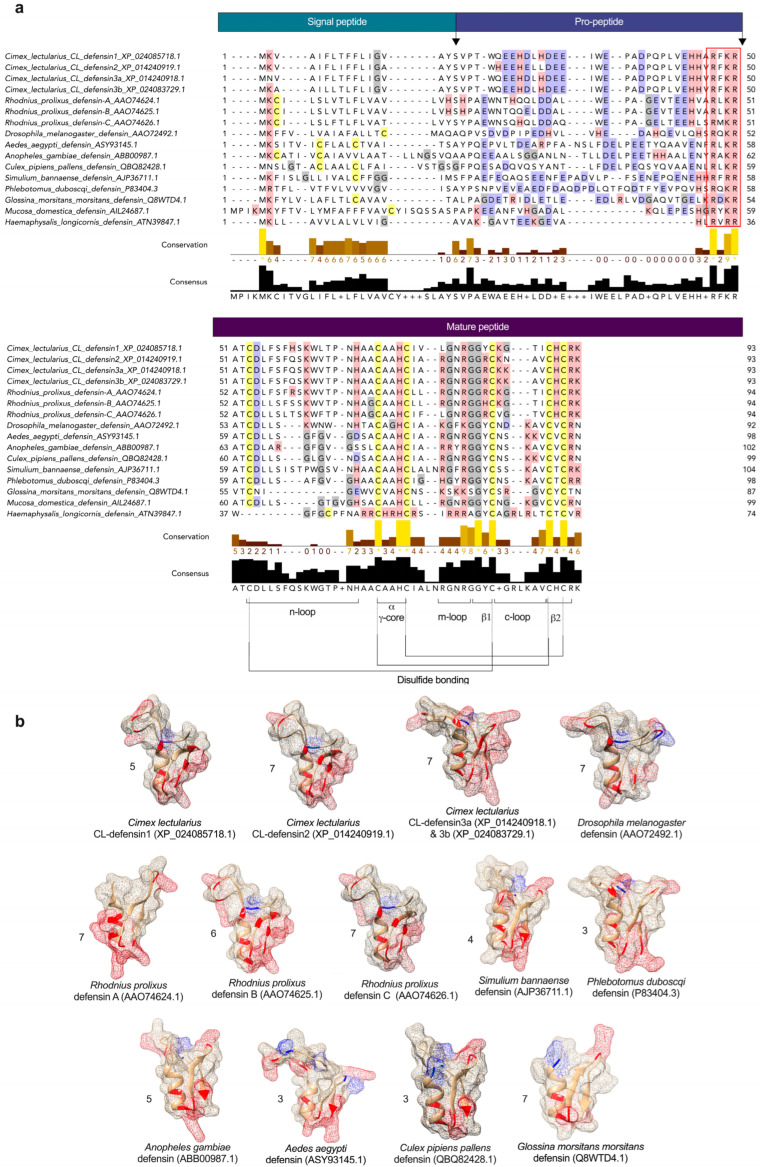
(**a**) Comparison of structural features of defensins in bed bugs, *Cimex lectularius*, and other select arthropods. Defensins were aligned using both MUSCLE (multiple sequence comparison by log-expectation; https://www.ebi.ac.uk/Tools/msa/muscle/, accessed on 26 January 2022) and multiple sequence alignment (MSA). Conserved cysteines are yellow-highlighted, positively charged side groups (basic residues; lysine (K), arginine (R), histidine (H)) are red-highlighted, and negatively charged side groups (acidic residues; glutamate (E), aspartate (D)) are blue-highlighted. The predicted signal-peptide, pro-peptide, and mature peptide regions are indicated in boxes above MSA and the cleavage sites between these regions are indicated by arrows. Conserved cysteines and glycines are yellow- and grey-highlighted, respectively. Conserved structural features, including the n-loop, α γ-core, m-loop closer to the numbers, β1, c-loop, β2, and the disulfide bridges, are all presented at the bottom of the figure. The RXXR mature peptide cleavage site motif is outlined by the red rectangle. (**b**) Comparison of predicted 3D structures of defensins from bed bugs and other select insects (using the protein structure homology-modelling server SWISS-MODEL), and the effect of amino acid substitution on predicted surface charge. Amino acids with basic residues (K, R, H) and acidic residues (E, D) are red- and blue-highlighted, respectively. The net charge of each defensin is represented next to its structure. Three of the bed bug defensins have a charge (5, 7) comparable to the charge (6, 7) of kissing bug, *Rhodnius prolixus*, defensins. Bed bug and kissing bug defensins, unlike defensins from other hematophagous arthropods and the vinegar fly *Drosophila melanogaster* have a stronger positive surface charge that interacts with the negative charge on microbial membranes, leading to their depolarization, perforation, and death [47]. The tertiary structures were predicted based on the template 1ica.1.A (Protein Data Bank ID)—3D structure of defensin MGD-1 from blow fly larvae, *Phormia terranovae* [48]. We used 1ica.1.A as a template because (*i*) it was highly ranked for all bed bug defensins, and (*ii*) the redefined 3-dimensional model of defensin A is derived from structural analyses using extensive two-dimensional nuclear magnetic resonance spectroscopy (786 inter-proton nuclear Overhauser effects) [48]. Sequence similarity between 1ica.1.A and the selected defensins was above 65% in all cases. The structures presented were derived from peptides in aqueous solution.

**Figure 2:**
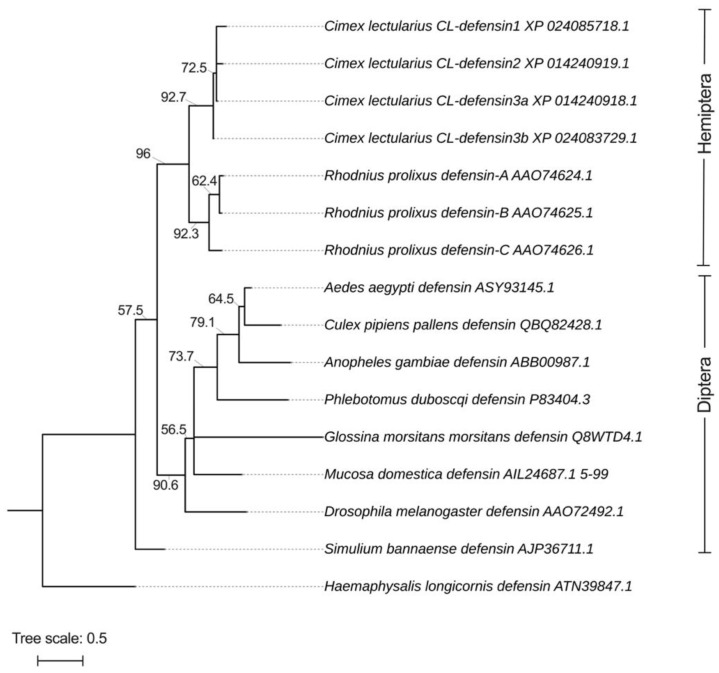
Phylogeny of the mature and active region of defensins identified in bed bugs, *Cimex lectularius*, and other select arthropods. The defensins isolated from bed bugs are most closely aligned with those from another hemipteran, the kissing bug *Rhodnius prolixus*. Defensin sequences were aligned with MUSCLE (https://www.ebi.ac.uk/Tools/msa/muscle/, accessed on 26 January 2022), and the alignments were used to build phylogenetic trees using iqtree-2.0-rc2 with substitution models BLOSUM62 and PMB. The tree was finalized using iTOL v6 (https://itol.embl.de/, accessed on 26 January 2022, Heidberg, Germany). Branch lengths are represented on top of each branch. Phylogenetic testing included 10,000 replicates of Ultrafast bootstrap (UFBoot) represented on each branch to provide support for tree branches. The defensin from the tick *Haemaphysalis longicornis* (ATN39847.1) was used as the outgroup.

We characterized the sequences of two previously uncharacterized defensin-like peptides and named them CL-defensin1 (XP_024085718.1) and CL-defensin2 (XP_014240919.1). We also characterized the sequences of two new defensin isoforms, CL-defensin3a (XP_014240918.1) and CL-defensin3b (XP_024083729.1) (Figure 1a). The complete amino acid sequence of these CL-defensins is strongly conserved (>87%), with minor differences only in the signal and pro-peptide regions. The mature peptide region of CL-defensin3a and CL-defensin3b is 100% conserved, with two amino acid differences only in the signal and pro-peptide regions. CL-Defensin1 has a net charge of +5, whereas other bed bug defensins have a net charge of +7. The lower charge of CL-defensin1 is caused by an additional histidine in position 9, and the absence of the conserved basic residues arginine in positions 27 and 33, and lysine in position 36 of the mature sequence. Comparing the charge of the mature defensins from various insect taxa (Figure 1b) revealed that kissing bug (*Rhodnius prolixus*) and bed bug (*Cimex lectularius*) defensins have a higher charge than mosquito (*Aedes aegypti*, *Anopheles gambiae*, *Culex pipiens*), black fly (*Simulium bannaense*), and sand fly (*Phlebotomus duboscqi*) defensins, due to the composition of their basic residues (K, R, H) and acidic residues (E, D). In a homology search, the mature region of bed bug defensins share an amino acid sequence identity of ~86% with defensin genes of triatomine bugs (*Panstrongylus megistus* (AHY02937.1), *Meccus pallidipennis* (AXY04223.1), and *R. prolixus* (AAO74624.1)). Whereas there is a strong conservation of the mature regions of defensins from the insects selected (Figure 1a), there is far less conservation in the signal peptide and pro-peptide regions. Phylogenetic analyses of these sequences indicate that bed bug defensins are most closely related to the *R. prolixus* defensins A and B (AA074625.1) (Figure 2).

### 2.2. Time-Dependent Defensin Expression Prompted by Bacterial Injection (Figure 3)

**Figure 3:**
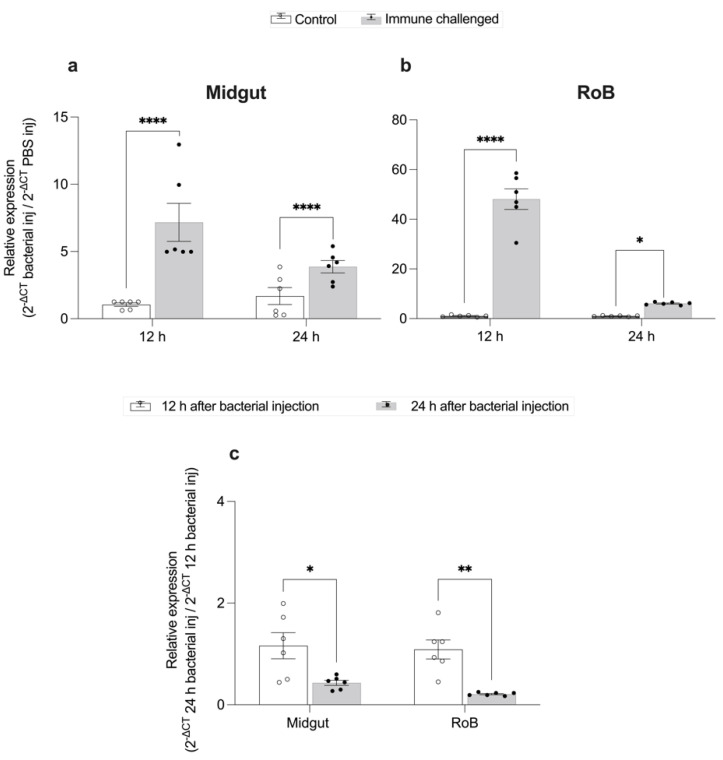
Time-dependent expression of CL-defensin1 mRNA (LOC106661793) in bed bugs after bacterial injection. Samples of midgut and RoB (rest of body containing bodies minus heads and midgut tissues) were collected from bed bugs 12 and 24 h after they were injected with a mixture of Gram-positive (*Bacillus subtilis* ATCC 6633) and Gram-negative (*Escherichia coli* K12/D31) bacteria. (**a**) Levels of defensin mRNA in the midgut 12 h and 24 h after bacterial injection. (**b**) Levels of defensin mRNA in the RoB 12 h and 24 h after bacterial injection. (**c**) Comparison of the effect of time on the defensin mRNA expression in the midgut and RoB 12 h and 24 h after bacterial injection. The relative expression of defensin was evaluated using the ΔΔCT method [49,50]. The expression level from PBS-control-injected samples was used as the second calibrator and set arbitrarily at 1 (subpanels a and b), and data at the 12-h time point (white bars) were used as the second calibrator in panel c. In panel c, the effect of time (12 h or 24 h) on defensin expression was compared using the formula 2^−^^ΔCt^ 24 h bacterial inj/2^−^^ΔCt^ 12 h bacterial inj. The data representing 12 h after bacterial injection were arbitrarily set to 1. The fold-change of defensin expression 24 h after bacterial injection is shown as grey bars. Bars represent the mean transcript levels ± 95% CI. Means were compared using the unpaired Student’s *t*-test (* *p* < 0.05, ** *p* < 0.01, **** *p* < 0.0001).

After bacterial injection of bed bugs with Gr+ and Gr– bacteria, CL-defensin1 in the midgut was upregulated 6-fold at 12 h (*p* < 0.0001) and 3-fold at 24 h (*p* < 0.0001) (Figure 3a). Similarly, CL-defensin1 in the RoB was upregulated 47-fold at 12 h (*p* < 0.01) and 9-fold at 24 h (*p* < 0.05) (Figure 3b). Between 12 and 24 h post bacterial injection, CL-defensin1 was downregulated 4-fold in the midgut (*p* < 0.01) and 2-fold in the RoB (*p* < 0.05) (Figure 3c). Based on these data, we selected 12 h post bacterial injection to evaluate the level of bed bug AMP defensin gene expression.

### 2.3. Midgut- and RoB-Distinct Gene Expression of CL-Defensins by Male and Female Bed Bugs Fed Bacteria-Infected Blood or Injected with Bacteria (Figure 4 and Figure 5)

**Figure 4:**
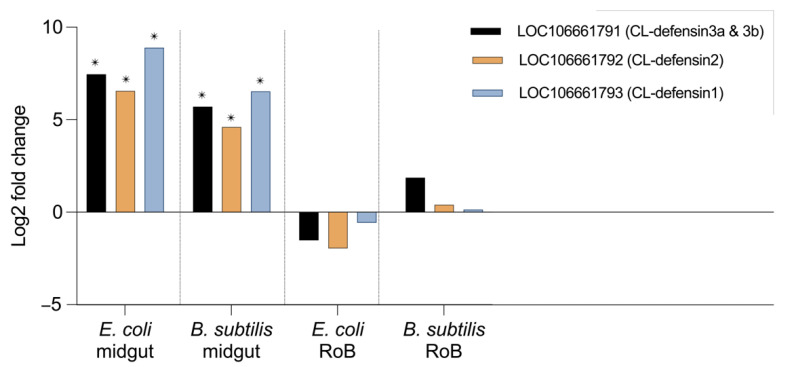
Comparative transcriptome (RNAseq) analyses of CL-defensin1, CL-defensin2, and CL-defensin 3a and 3b expression in bed bugs after ingestion of bacteria-infected blood. Comparative analyses of gene expression in the transcriptome study in bed bug midguts and RoB tissues (rest of body containing bodies minus heads and midgut tissues) after ingestion of sterile blood or blood infected with the Gram-positive bacterium *Bacillus subtilis ATCC 6633* or the Gram-negative bacterium *Escherichia coli* K12/D31. The Wald test was used to generate *p*-values and Log2 fold changes. An asterisk indicates statistically significant changes in gene expression levels (adjusted *p*-values < 0.05).

**Figure 5:**
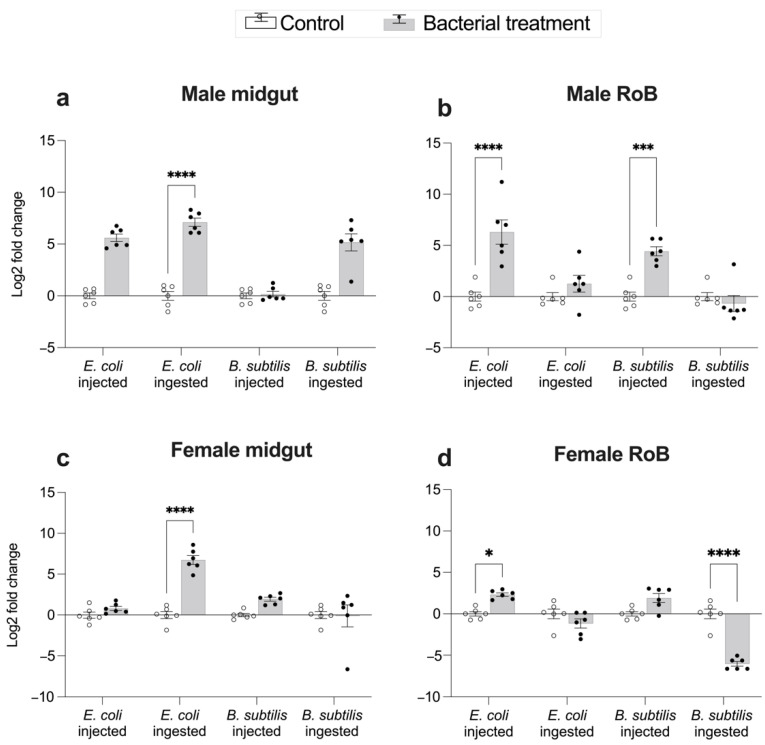
Changes in expression levels of CL-defensin1 in the midguts and RoB (rest of body containing bodies minus heads and midgut tissues) of male (**a**,**b**) and female (**c**,**d**) bed bugs 12 h after intrathoracic injection or ingestion of Gram-negative (*Escherichia coli* K12/D31) or Gram-positive (*Bacillus subtilis* ATCC 6633) bacteria. White bars represent data obtained from control bugs that were injected with phosphate buffer saline (PBS) or that ingested sterile blood. The relative expression of CL-defensin1 (LOC106661793) was evaluated using the ΔΔCT method [49,50]. Bars represent the mean transcript levels ± 95% CI. Means were compared using the unpaired Student’s *t*-test (* *p* < 0.05, *** *p* < 0.001, **** *p* < 0.0001).

Comparative transcriptomics revealed differences in midgut- and RoB-upregulation of CL-defensins (Figure 4). All defensin genes (LOC106661793 encoding CL-defensin1, LOC106661792 encoding CL-defensin2, and LOC106661791 encoding CL-defensin3a and CL-defensin3b) were significantly upregulated in the midgut after ingestion of blood infected with Gr– *E. coli* or Gr+ *B. subtilis* (Figure 4). In the RoB, however, none of the defensin genes was significantly upregulated (Figure 4). Due to high sequence similarity, only CL-defensin1 was further evaluated using qPCR and specific primers. Comparative transcriptomics results are consistent with the qPCR results of CL-defensin1 expression in the midgut and the RoB (Figure 5).

qPCR results illustrated that in the midgut of male bed bugs, the expression of CL-defensin1 (LOC106661793) increased 165-fold after a blood meal containing Gr– bacteria (*p* < 0.0001) but did not significantly increase (*p* > 0.05) after a blood meal containing Gr+ bacteria, or after intrathoracic injections of Gr– bacteria or Gr+ bacteria (Figure 5a). In the RoB of male bed bugs, the expression of this defensin did not significantly increase (*p* > 0.05) after a blood meal containing either Gr– or Gr+ bacteria, but increased 452-fold after intrathoracic injection of Gr– bacteria (*p* < 0.0001), and increased 26-fold after intrathoracic injection of Gr+ bacteria (*p* < 0.001) (Figure 5b).

In the midguts of female bed bugs, the expression of CL-defensin1 increased 149-fold after a blood meal containing Gr– bacteria (*p* < 0.0001) but did not significantly increase (*p* > 0.05) after a blood meal containing Gr+ bacteria, or after intrathoracic injection of Gr– or Gr+ bacteria (Figure 5c). In the RoB of female bed bugs, the expression of CL-defensin1 (LOC106661793) did not increase after a blood meal containing Gr– bacteria, decreased 67-fold after a blood meal containing Gr+ bacteria (*p* < 0.0001), and increased 5-fold after intrathoracic injection of Gr– bacteria (*p* < 0.05) but did not significantly increase after intrathoracic injection of Gr+ bacteria (Figure 5d).

### 2.4. Effect of Bed Bug Sex and Mode of Immune Challenge on the Upregulation of CL-Defensins (Figure 6 and Figure 7)

**Figure 6:**
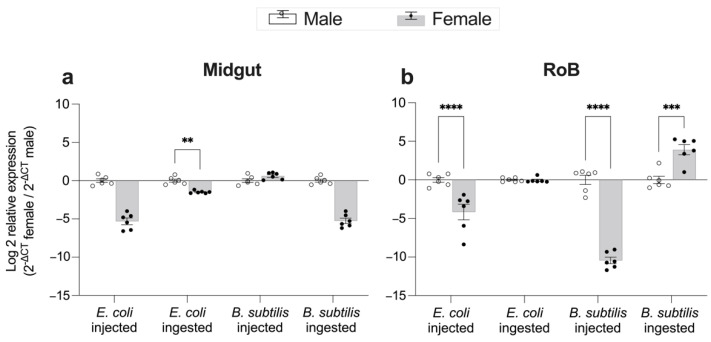
Effect of bed bug sex on changes in expression levels of CL-defensin1 12 h after intrathoracic injection or ingestion of the Gram-positive bacterium *Bacillus subtilis* (ATCC 6633) or the Gram-negative bacterium *Escherichia coli* (K12/D31) in the midgut (**a**) and RoB (rest of body containing bodies minus heads and midgut tissues) (**b**). The relative expression of defensin was evaluated using the ΔΔCT method [49,50]. Data from males were used as the second calibrator and were arbitrarily set to 1; the fold-changes of defensin expression in females are shown as grey bars. Bars represent the mean transcript levels ± 95% CI. Means were compared using the unpaired Student’s *t*-test (** *p* < 0.01, *** *p* < 0.001, **** *p* < 0.0001).

Gene expression of CL-defensin1 in the midgut and RoB was generally higher in male than in female bed bugs after either ingestion of bacteria-infected blood or intrathoracic injection of bacteria (Figure 6). Ingestion of Gr+ bacteria, however, caused significantly higher expression of CL-defensin1 in the RoB of females than males (Figure 6b).

**Figure 7:**
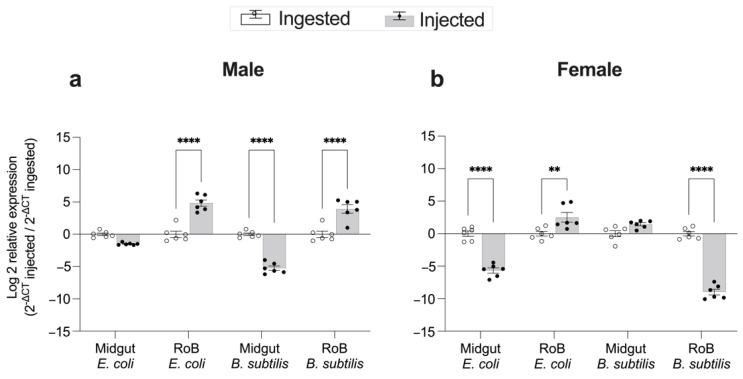
Comparison of the mode of infection, bacterial ingestion, or injection, on changes in expression levels of CL-defensin1 in bed bugs. Samples of midgut and RoB (rest of body containing bodies minus heads and midgut tissues) were collected from male (**a**) and female (**b**) bed bugs 12 h after the injection of bacteria (Gram-positive *Bacillus subtilis* ATCC 6633 or Gram-negative bacteria *Escherichia coli* K12/D31), or 12 h after ingestion of blood infected with *E. coli* or *B. subtilis*. The relative expression of defensin was evaluated using the ΔΔCT method [49,50]. The effect of bacterial ingestion versus bacterial injection was compared using the formula 2^−^^ΔCt^ injected/2^−^^ΔCt^ ingested. The ingestion-sample data (white bars) representing the calibrator were arbitrarily set to 1, and fold-changes in defensin expression in bacterial injection sample data are shown as grey bars. Bars represent the mean transcript levels ± 95% CI. Means were compared using the unpaired Student’s *t*-test (** *p* < 0.01, **** *p* < 0.0001).

The effect of bacteria injection or ingestion on expression levels of CL-defensin1 in female and male bed bugs is shown in Figure 7. In males, injections of *E. coli* and *B. subtilis* caused 37-fold and 21-fold, respectively, higher expression of CL-defensin1 in the RoB than did ingesting bacteria (*p* < 0.0001) (Figure 7a). In the midgut of males, the mode of immune challenge (injection vs. ingestion) with *E. coli* had no differential effect on gene expression, but *B. subtilis* injection caused 33-fold lower gene expression than ingestion (*p* < 0.0001). Female bed bugs exhibited different responses than males. In the RoB of females, *E. coli* injection caused 11-fold higher gene expression than *E. coli* ingestion (*p* < 0.001) but *B. subtilis* injection caused 400-fold lower gene expression then *B. subtilis* ingestion (*p* < 0.0001) (Figure 7b). In the midgut of females, *E. coli* injection caused 50-fold lower gene expression than *E. coli* ingestion (*p* < 0.0001), whereas the delivery method (injection vs. ingestion) with *B. subtilis* had no differential effect on defensin expression (Figure 7b). 

### 2.5. Effect of Blood Ingestion on the pH of Bed Bug Midgut and RoB (Figure 8)

There was no difference in the mean midgut pH (5.74 vs. 5.65) of bed bugs that did, or did not, ingest blood after 28 days of starvation. Conversely, the mean RoB pH of bed bugs decreased from 6.63 to 6.28 (*p* < 0.001) 24 h after a blood meal. In both blood-fed and control bed bugs, midgut pH was lower than the RoB pH (*p* < 0.0001 each). 

## 3. Discussion

Insects possess multiple innate immune signaling pathways that respond to microbial invasions. The activation of these pathways results in the expression of potent AMPs as one component of the insects’ systemic immune responses [29]. Once the microbial ligands are recognized, the Toll and IMD pathways generally become the most important intracellular immune signaling mechanisms for AMP expression [51,52]. Our current knowledge of insect AMP regulation stems mostly from studies on holometabolous insects such as *D. melanogaster* and *Ae. aegypti* which creates a dipteran bias. However, although hemimetabolous insects have functional immune systems that share many conserved components with holometabolous insects, in some hemimetabolous insects, including bed bugs and kissing bugs, there are deviations from the standard dipteran models [53,54,55]. Even insects that are close phylogenetic relatives may have evolved different AMP spectra and expression profiles, probably due to the type of microbes to which they have been exposed through evolutionary time [10,56,57]. In contrast to vectors that routinely transmit pathogens and parasites, bed bugs—despite their hematophagous feeding behavior—are believed not to transmit disease-causing agents to vertebrates. The underlying mechanisms for the bed bugs’ vector incompetence are not well understood but may comprise a strong innate immune system, including AMPs, and possibly novel yet-to-be identified factors. 

Defensins are common AMPs expressed in most invertebrates. We evaluated the structures and phylogenetic associations of bed bug defensins and illustrated the role they play in midgut immunity in bed bugs towards ingested bacteria. Oral ingestion and intrathoracic injection of Gr+ and Gr– bacteria differentially affected defensin expression patterns in midgut and RoB. We also show that male and female bed bugs have sex-specific defensin expression, which may be related to their peculiar mode of sperm transfer [58]. While we specifically studied transcripts for CL-defensins, we do not exclude the possibility that many more bed bug AMPs await discovery.

All of the defensins described here have the same predicted structures in the mature peptide: six cysteine residues and three disulfide bridges making up an N-terminal loop, an alpha-helix, and an antiparallel beta-sheet. Our in silico analysis of the complete amino acid sequence of all these bed bug defensins revealed high sequence similarity and a precursor organization to classical insect-type defensins (CITDs), comprising a signal peptide, and an acidic pro-peptide ending with a conserved cleavage site [29] to release the mature peptide (Figure 1a).

We did not detect any putative glycosylation sites in any of the bed bug defensin sequences. However, for CL-defensin2, CL-defensin3a, and CL-defensin3b, we did detect putative phosphorylation sites at positions 52 (threonine-specific), 57 (serine-specific), 60 (serine-specific), and 64 (threonine-specific). CL-defensin1 had three potential phosphorylation sites, with position 60 not being phosphorylated. Reversible phosphorylation is very important in protein-protein interactions via recognition domains because many proteins and receptors are switched “on” or “off” by phosphorylation and dephosphorylation [59]. Reversible phosphorylation may also result in conformational changes in the structure of peptides, causing them to become activated, deactivated, or degraded [60]. Furthermore, structural comparison of mature defensins (Figure 1b) demonstrates the distribution of basic residues (K, R, H) and acidic residues (E, D) that may affect the total charge of bed bug defensins. The net charge of bed bug defensins (CL-defensin1: 5; CL-defensin2: 7; CL-defensin3a and CL-defensin3b: 7 each) and of kissing bug defensins (6, 7) is higher than that of most other insect defensins. The higher charge of bed bug and kissing bug mature defensins (Figure 1b) may enhance the electrostatic attraction between the cationic defensins and negatively charged components present on the outer bacterial envelope [61], leading to the depolarization, insertion, and perforation of these membranes [47,62]. 

**Figure 8:**
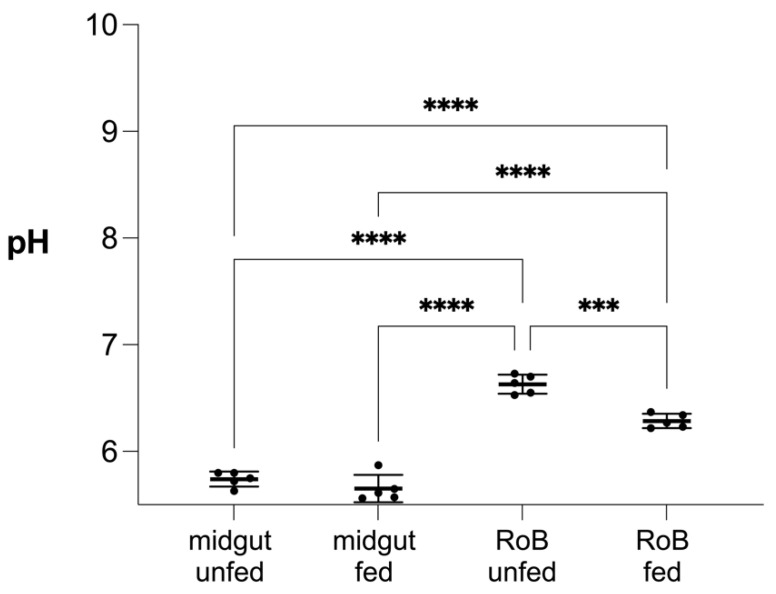
Effect of blood ingestion by male bed bugs on pH changes in their midgut and RoB (rest of body containing bodies minus heads and midgut tissues). Midgut and RoB tissue were dissected from >28-day-starved bed bugs that ingested blood (“fed”) or did not (“unfed”). Data were collected 24 h after blood ingestion. The pH was measured using the LAQUAtwin pH 22 pH meter (Horiba, Kyoto, Japan). At least five biological replicates were used per treatment or control group and five insects were pooled for each group. Data were analyzed by one-way ANOVA and Brown–Forsythe test (*** *p* < 0.001, **** *p* < 0.0001).

The antimicrobial activity of defensins is also affected by the chemical properties of their environment and must be assessed in the context of the physiological conditions encountered in the insect body such as the pH or salt concentration. The pH of the bed bug midgut and RoB affects the charge of bed bug defensins and, in turn, their antimicrobial activity or ability to bind to bacterial membranes [47]. The pH in the bed bug midgut (5–6) and hemolymph (6–7) (Figure 8) is well within the pH range (5–7) which enables optimal antimicrobial activity of defensins [63]. In the kissing bug, *Triatoma infestans*, general AMP activity is stronger at pH 5 than at pH 7 [11]. In response to dissimilar pH in the gut and hemolymph, insects have likely evolved immune peptides to work optimally in their respective tissues [63]. This may explain the expression of multiple defensins with different charges and activities in different tissues of the same insect. Insect defensins help eliminate microbes and may also have an immune signaling function [64], which would affect our interpretation of high levels of transcripts after microbial ingestion or injection. Microbes attempting to colonize insect hosts face many challenges including acid stress (inhospitably low pH) upon entry into the host [65]. The digestion of blood produces high levels of heme and reactive oxygen intermediates that are toxic to both insect and microbe [66]. Detoxifying these compounds will affect pH and other physiological factors that any pathogen must tolerate. 

### 3.1. Induction of Immune Pathway Effector AMPs in Response to Gr– and Gr+ Bacteria in the Midgut and RoB

We have demonstrated an induction of defensin expression in bed bug midguts and RoB tissues after the ingestion of blood infected with Gr+ or Gr– bacteria. The data indicate a regulatory function of CL-defensins in eliminating pathogens that bed bugs may ingest during blood meals. 

Defensins may be co-regulated by both IMD and Toll pathways [23]. “Functional permeation” [23] and cross-talk of these pathways have already been demonstrated in other hemipterans [12,23]. Intrathoracic injections of Gr+ or Gr– bacteria induced similar upregulation of the same AMPs [23], and RNA interference (RNAi) of the IMD pathway suppressed the upregulation of effector molecules in both Toll and IMD pathways [23,24]. In the stink bug *Plautia stali*, intrathoracic injection of both Gr+ and Gr– bacteria caused the upregulation of effector genes in both pathways [23]. Our findings that bed bugs upregulate CL-defensins in response to challenges with both Gr+ and Gr– bacteria provide further evidence for functional cross-talk and blurred functional differentiation between the Toll and the IMD pathways [67]. How this functional cross-talk occurs, and which recognition molecules are used in multiple pathways is yet unknown. 

Defensin expression was strongest 12 h after injection treatment and was lower at 24 h after treatment in both midgut and RoB tissues. This transient upregulation of defensins in bed bugs resembles the inducible and transient expression of many AMPs in different insects in response to microbial challenges [10,68,69,70]. The timing and duration of AMP expression may be affected by the number and the location of pathogens and parasites that induce the immune responses. In *R. prolixus*, both AMP expression and antimicrobial activity are highest seven days after infection with the parasite *Trypanosoma rangeli* [45]. In bed bugs, antimicrobial activity in midgut and RoB tissues was already higher 8 h after injections of Gr+ and Gr– bacteria and was even higher at 24 h [67]. Significant upregulation of CL-defensins 12 h after bacterial injections may have contributed to the increased AMP activity 24 h after bacterial injection [67] along with other antimicrobial factors. 

### 3.2. Effects of Bed bug Sex and Mode of Immune Challenge on Upregulation of AMPs

The bed bugs’ unusual reproductive strategy of traumatic insemination [58] causes copulatory wounding in females and increases their risk of microbial infections. As females typically are mated after a blood meal, they have developed reproductive immune anticipation triggered by feeding cues [71] to deal with any microbes that are injected with sperm or that otherwise invade their bodies. In response to feeding cues and preceding traumatic insemination, females pre-emptively express AMPs and upregulate lysozyme-like activity [17,58,71,72,73]. As previously reported, blood-fed female bed bugs have stronger overall antimicrobial activity than blood-fed male bed bugs [67]. Here we studied whether the mode of immune challenge affects the upregulation of defensins in a sex-specific manner. After ingestion and injection of bacteria-laced blood, males had the highest defensin expression in their midgut and RoB, respectively. Defensin expression by females, however, did not follow this pattern and ingestion of Gr+ bacteria caused a higher response in the RoB. Regardless, our data show that the mode of immune challenge affects sex-specific defensin expression in bed bugs.

In dipterans, defensins are most strongly active against Gram+ bacteria, fungi, and some protozoan parasites [17,34]. However, our results demonstrate defensin expression against both Gram-positive and Gram-negative bacteria, consistent with defensin responses in hemimetabolous insects that show activity against both Gram-positive and Gram-negative bacteria [42]. In addition, defensin peptides play other vital roles in other aspects of the immune response, such as functioning as signaling molecules or as a stress response element when pathogens in the hemolymph exceed the phagocytic capacity [64]. Thus, the increase in defensin expression may also serve this role in bed bugs.

Bed bugs, and many other hemimetabolous insects, must be able to recognize and eliminate potential pathogens without eliminating essential bacterial symbionts on which they rely for their very survival. These symbionts, including *Wolbachia* spp. and an α-proteobacterium [74,75,76,77], help bed bugs obtain essential nutrients not present in vertebrate blood. When *Wolbachia* spp. symbionts are reduced in number or eliminated experimentally, the development of bed bug nymphs was hindered or slowed, and adults were smaller and less fit [78]. Not surprisingly, insects have evolved mechanisms to protect their symbionts against harmful host immune responses [79]. For example, some aphid species reduce the repertoire of their immune receptors or produce more specific AMPs around their bacteriome to prevent the escape and elimination of their symbionts [37,80,81]. Similar adaptations have been reported in holometabolous insect–endosymbiont systems [82]. Furthermore, in blood-fed *Rhodnius* spp. and *Triatoma* spp., bacterial populations in the anterior and posterior midgut significantly increase concurrently with the downregulation of host insect immune factors [45,66,83]. Once host immune factors are upregulated, bacterial populations decline and these bacteria are digested, implying a supplementary nutritional role of these symbionts [83,84]. The effects of immune responses by bed bugs on their endosymbionts and microbiome, and the fine line between succumbing to bacterial infections, regulating a balanced microbiome, and avoiding elimination of beneficial symbionts have not yet been elucidated. 

In conclusion, we characterized two new defensin AMPs, and two new defensin isoforms in bed bugs, and determined their molecular features, structural properties, and phylogenetic relatedness to other insect defensins. We evaluated the expression of defensins in the midgut and RoB tissues after bacterial immune challenges. Using comparative transcriptomics and qPCR, we found sex-specific and immune challenge-specific (bacterial ingestion vs. bacterial injection) induction of CL-defensins. The activity of these AMPs should be evaluated against other parasites and pathogens to determine their effectiveness under different physiological conditions in different body regions or tissues of the insects. Future studies should also test the immune responses of bed bugs after ingestion or injection of medically relevant human pathogens to understand how bed bugs respond to these pathogens with the goal of determining those specific factors that might explain why bed bugs are not vectors of parasites and pathogens, in contrast to almost all other hematophagous arthropods. 

## 4. Materials and Methods

### 4.1. Laboratory Rearing of Bed Bugs

Colonies of bed bugs were maintained as described previously [85]. Briefly, colonies were kept in the insectary of Simon Fraser University (SFU) at a temperature of ∼24 °C, ambient relative humidity, and a photoperiod of 14 h light to 10 h dark. Groups of 150 bed bugs were maintained in 50-mL glass jars fitted with a square of cardboard (2 cm × 2 cm) at the bottom and a strip of cardboard (2 cm × 4 cm) diagonally across the jar. Bed bugs in separate jars were fed on the forearm of a volunteer (Regine Gries) once every month. For feeding, jars were covered with fine mesh, inverted, and pressed against the forearm so that the bed bugs could feed through the mesh.

### 4.2. Growth and Preparation of Bacteria and Immune Challenges of Bed Bugs

Microbes (*E. coli* (K12/D31) and *B. subtilis ATCC 6633*) were grown in separate Lysogeny Broth (LB) [86] for 17 h at 30 °C in a shaking incubator (220 revolutions per minute). Subsequently, the bacteria were reinoculated into fresh broth and incubated for 4 h under the same conditions to reach the log phase of growth. The bacteria were then washed three times in sterile phosphate buffered saline (PBS; 0.01 M phosphate buffer, 2.7 mM potassium chloride, 0.137 M sodium chloride, pH 7.4), and were diluted either in sterile defibrinated rabbit blood or in PBS to a final concentration of ~1 × 10^6^ cells/mL.

Bed bugs were exposed to bacteria orally (ingestion of microbe-infected blood) or through intrathoracic injection of bacteria directly into the hemocoel. For oral infections, we deployed a water-jacketed membrane feeder (Thermo Fisher Scientific Isotemp 2150 B14, USA) maintained at 37 °C with stretched out parafilm as the membrane. Male or female bed bugs that had not been fed for >20 days were allowed to feed for 1 h on defibrinated rabbit blood containing bacteria (*E. coli* or *B. subtilis*). Control bed bugs ingested sterile blood. Fully engorged bed bugs were separated and housed in glass jars until analysis. For intrathoracic injections, we injected 0.5 μL of bacteria (*E. coli*, *B. subtilis*, or both at a final concentration of approximately 1 × 10^6^ cells/mL) or a PBS control into the hemocoel of male or female bed bugs that had not been fed for >20 days. Each experiment had five replicates, with five insects each in treatment and control samples.

### 4.3. Tissue Isolation and RNA Extraction

Midgut and rest of body tissues (RoB; containing bodies minus the heads and midgut tissues) of treatment and control bed bugs were dissected 12 h and 24 h after the immune challenge, and total RNA was extracted using TRizol reagent (Invitrogen) following the manufacturer’s recommendations. The samples were quantified on a Nanodrop 1000 spectrophotometer v. 3.7 (Thermo Fisher Scientific, USA). RNA samples were used to generate cDNAs for analysis using quantitative real-time PCR (qPCR) and transcriptome assembly.

### 4.4. cDNA Synthesis and Quantitative Real-Time PCR (qPCR)

First strand cDNA synthesis was performed in 20-μL reaction mixtures containing 2.0 μg total RNA using a modified oligo dT primer (MgdT [44]) with the OneScript cDNA Synthesis Kit (ABM, CA) and an extension time of 50 min. The subsequent cDNA was diluted (1:10) with sterile molecular grade RNase-free water.

The expression of transcripts of CL-defensin1 in the different samples was assessed using qPCR. All qPCR reaction mixtures contained 2 μL of cDNA, 300 μM of each primer, and 5 μL of PerfeCTa SYBR Green Super Mix (Quanta Biosciences, USA) in a final volume of 10 μL. qPCR was performed on a LightCycler96 thermal cycler (Roche Diagnostics, DE), with the following conditions: 95 °C (5 min), 40 cycles each at 95 °C (15 s) and 60 °C (40 s), followed by a melt curve analysis to confirm the specificity of reactions. No-template controls were included with each primer set to verify the absence of exogenous DNA and primer-dimers. Each primer pair had 99% efficiency as determined using the slope of a linear regression model (SM, unpubl. data). The primer sequences are reported in Table 1. Primers were designed or retrieved from the literature for (*i*) ribosomal protein (RPL18) as the internal control gene, which provides the most stable gene expression across all tissues and developmental stages of bed bugs [87], and (*ii*) CL-defensin1. The amplicons were sequenced to confirm they had amplified the correct sequence. Because the sequences are so similar, CL-defensin1 was the only sequence we could amplify individually.

### 4.5. qPCR Analysis

Relative differences in transcript levels were calculated using the DELTA (Δ) threshold cycle (CT) method 2^–^^ΔΔCT^ [49,50]. We normalized expression levels using an internal control gene (RPL18) and to generate ΔCT values. In the 2^−^^ΔΔCT^ method, we used control samples (bed bugs injected with a PBS control or fed sterile blood) as the second calibrator to measure fold changes in expression levels (Figure 3a,b; Figure 5). We compared time-dependent (12 h vs. 24 h) expression levels of defensin transcripts (CL-defensin1 (LOC106661793)) in the midgut and RoB of male bed bugs, using the formula 2^−^^ΔCT^ 24 h following bacterial injection/ 2^−^^ΔCT^ 12 h following bacterial injection (Figure 3c). We compared sex-dependent (female vs. male bed bugs) gene expression of CL-defensins, using the formula 2^−^^ΔCT^ female/2^−^^ΔCt^ male (Figure 6), and we compared the effects of intrathoracic bacterial injection and oral bacterial ingestion, using the formula 2^−^^ΔCt^ injected/2^−^^ΔCt^ ingested (Figure 7). The results are presented as means and standard errors of at least three independently generated cDNAs assayed at least twice, with each sample run in three technical replicates. All data sets were tested for normality using the Shapiro–Wilk normality test and were compared using the unpaired Student’s *t*-test or Mann–Whitney U test, when appropriate. Prism version 9.4.1 software (GraphPad Software, San Diego, CA, USA) was used for statistical analyses and 2D graphing. The statistical significance level was 0.05. Relative transcript levels are expressed as means with whiskers representing ± SEM (* *p* < 0.05, ** *p* < 0.01, *** *p* < 0.001, **** *p* < 0.0001).

### 4.6. Transcriptome Assembly: Library Preparation with PolyA Selection and HiSeq Sequencing and RNA-Seq Data Analysis

We created a de novo transcriptome assembly from the RNA samples that we extracted from midgut and RoB tissues of male bed bugs 12 h after ingesting a blood meal containing *E. coli* or *B. subtilis*. RNA purification, first and second strand synthesis, adaptor ligation, quantification, validation, and Illumina sequencing were all done at GENEWIZ LLC. (South Plainfield, NJ, USA).

The samples were sequenced using a 2 × 150 bp paired-end (PE) configuration. Image analysis and base calling were conducted using HiSeq Control Software (HCS). Raw sequence data (.bcl files) generated from Illumina HiSeq were converted into fastq files and demultiplexed using bcl2fastq 2.17 software (Illumina Inc, San Diego, CA, USA). One mismatch was allowed for index sequence identification. After investigating the quality of the raw data, sequence reads were trimmed using Trimmomatic v.0.36 to remove adapter sequences and nucleotides with poor quality. The trimmed reads were mapped to the reference genome available on ENSEMBL using STAR aligner v.2.5.2b that uses a splice aligner to detect splice junctions and incorporates them to help align the entire read sequences to generate BAM files. We calculated the unique gene hit counts using the Counts feature from the Subread package v.1.5.2, counting only unique reads that fell within exons.

We used the gene hit counts table for downstream differential expression analysis using DESeq2 [88] to compare gene expression between groups of samples. The Wald test was used to generate *p*-values and Log2 fold-changes. Genes with adjusted *p*-values < 0.05, and with absolute log2 fold-changes >1, were considered to be differentially expressed genes. The data for the defensin transcripts were mined to evaluate their expression level and were reported as transcripts per million (TPM).

### 4.7. AMP Characteristic Identification

The full-length peptide sequences for bed bug defensins were retrieved from (https://www.ncbi.nlm.nih.gov/, accessed on 26 January 2022). All defensin amino acid sequences were submitted to the SignalP5.1 server (http://www.cbs.dtu.dk/services/SignalP/, accessed on 26 January 2022) to predict signal peptides. Pro-peptides were detected by ProP 1.0 Server (http://www.cbs.dtu.dk/services/ProP/, accessed on 26 January 2022). The net charge of the mature region of defensins at pH 7 and their molecular weights (MW) were predicted at PROTEIN CALCULATOR v3.4 server (http://protcalc.sourceforge.net/, accessed on 26 January 2022) (supplementary Table S1). The potential antimicrobial properties of the mature region of peptides were predicted in the Collection of the Anti-Microbial Peptides (CAMP_R3_) server (http://www.camp.bicnirrh.res.in/prediction.php, accessed on 26 January 2022) (Supplementary Table S1). Sequence similarity searches for obtained sequences were run with Blast, using the database of NCBI (http://blast.ncbi.nlm.nih.gov/Blast.cgi, accessed on 26 January 2022). Predicted tertiary structures of the mature or active defensin peptides (Figure 1b) were based on the template 1ica.1.A (PDB ID), the 3D structure of defensin MGD-1 from insect defensin A [48] predicted by SWISS-MODEL (https://swissmodel.expasy.org, accessed on 26 January 2022). Sequence similarities between 1ica.1.A and insect defensins (all defensin structures are presented in Figure 1b) were all above 50%. The tertiary structure models were visualized using UCSF Chimera [89].

In silico analyses to identify potential sites for post translational modifications, including glycosylation and phosphorylation, were performed using tools from the Center for Biological Sequence Analysis (http://www.cbs.dtu.dk/services/, accessed on 26 January 2022).

### 4.8. Phylogenetic Analysis

Multiple complete sequences, or conserved domain sequences, were aligned with MUSCLE aligner (https://www.ebi.ac.uk/Tools/msa/muscle/, accessed on 26 January 2022) using Jalview [90]. Alignments were used to build phylogenetic trees using iqtree-2.0-rc2 by selecting the best-fit substitution models BLOSUM62 and PMB. Maximum likelihood analyses were done using IQ-TREE v. 2.0 [91]. Best-fit models for each alignment were selected based on Bayesian information testing including 10,000 replicates of Ultrafast bootstrap (UFBoot) to provide support for tree branches. In our study, both BLOSUM62 and PMB models generated very similar results with good agreement. The tree presented here was prepared by iTOL v6 (https://itol.embl.de/, accessed on 26 January 2022, Heidberg, Germany). For the phylogenetic tree construction, the mature region of defensins from bed bugs, other hematophagous vectors, and the vinegar fly *Drosophila melanogaster* that have previously been tested for their activity against pathogens were utilized. The tick *Haemaphysalis longicornis* defensin (ATN39847.1) was used as the outgroup.

### 4.9. pH Measurements

Midgut and RoB tissues were obtained from >28-day-starved bed bugs that were, or were not (control), blood-fed 24 h prior to dissections. All tissues were homogenized in 200 μL of sterile water, and at least five biological replicates were processed for treatment and control samples, with five insects used for each replicate. pH values of midgut and RoB samples were determined with a pH meter (LAQUAtwin pH 22; Horiba, Kyoto, Japan) calibrated with sterile water (HPLC grade, EMD Millipore Corp., Burlington, MA, USA). Between measurements, the pH-meter was rinsed with water and regularly recalibrated with a pH 7 and a pH 4 buffer (Horiba, Kyoto, Japan). Prism version 9.4.1 (GraphPad Software, San Diego, CA, USA) was used for statistical analyses and 2D graphing. *p*-Values < 0.05 were considered significantly different. Data were analyzed with one-way ANOVA and the Brown–Forsythe test (* *p* < 0.05, ** *p* < 0.01, *** *p* < 0.001, **** *p* < 0.0001).

## Figures and Tables

**Table 1:** Primer sequences used for qPCR analyses.

Gene	Primer Sequence (5′–3′)
RPL18	FW: AAAGGCACGGTTACATCAAAGGTG RV: TAGTCTTGAACCTATAGGGGTCCC
CL-defensin1 (LOC106661793)	FW: AAG AGC GAC CTG CGA TTT GT RV: TGT GCC TTT ACA ATA CCC TCC TC

## Data Availability

Data are contained within the article or supplementary material.

## Supplementary Materials

The following supporting information can be downloaded at: https://www.mdpi.com/article/10.3390/ijms231911505/s1.
