# Supplementary material for: Characterization of New Defensin Antimicrobial Peptides and Their Expression in Bed Bugs in Response to Bacterial Ingestion and Injection
Source: Int J Mol Sci. 2022 Sep 29;23(19):11505. doi: 10.3390/ijms231911505 (PMC9570333; doi:10.3390/ijms231911505)
Supplement: Supplementary file 1 [file ijms-23-11505-s001.zip › ijms-1939633-supplementary.pdf]

Table S1. Physiological and predicted antimicrobial properties of defensins identified in the common bed bug, *Cimex lectularius*.

Peptide Name	Accession	Full Sequence			Mature Peptide	
		Molecular Mass (g/mol)	Mean Hydrophobicity	AMP Probability	Molecular Mass	Hydrophobicity
CL-defensin1	XP_024085718.1	10649.22	-0.19	0.984	4692.51	-0.32
CL-defensin2	XP_014240919.1	10658.35	-0.10	0.999	4690.45	-0.03
CL-defensin3a	XP_014240918.1	10675.29	-0.07	0.999	4704.53	0.03
CL-defensin3b	XP_024083729.1	10593.27	-0.01	0.999	4704.53	0.03
